# TASH: Toolbox for the Automated Segmentation of Heschl’s gyrus

**DOI:** 10.1038/s41598-020-60609-y

**Published:** 2020-03-03

**Authors:** Josué Luiz Dalboni da Rocha, Peter Schneider, Jan Benner, Roberta Santoro, Tanja Atanasova, Dimitri Van De Ville, Narly Golestani

**Affiliations:** 1 Brain and Language Lab, Department of Psychology, Faculty of Psychology and Educational Sciences, University of Geneva, Geneva, Switzerland; 2 Department of Neurology, Section of Biomagnetism, University Hospital Heidelberg, Heidelberg, Germany; 3 Department of Neuroradiology, University Hospital Heidelberg, Heidelberg, Germany; 4 Faculty of Psychology and Educational Sciences, University of Geneva, Geneva, Switzerland; 5 Medical Image Processing Lab, Institute of Bioengineering, École Polytechnique Fédérale de Lausanne, Lausanne, Switzerland; 6 Department of Radiology and Medical Informatics, University of Geneva, Geneva, Switzerland

**Keywords:** Cortex, Biomedical engineering

## Abstract

Auditory cortex volume and shape differences have been observed in the context of phonetic learning, musicianship and dyslexia. Heschl’s gyrus, which includes primary auditory cortex, displays large anatomical variability across individuals and hemispheres. Given this variability, manual labelling is the gold standard for segmenting HG, but is time consuming and error prone. Our novel toolbox, called ‘Toolbox for the Automated Segmentation of HG’ or TASH, automatically segments HG in brain structural MRI data, and extracts measures including its volume, surface area and cortical thickness. TASH builds upon FreeSurfer, which provides an initial segmentation of auditory regions, and implements further steps to perform finer auditory cortex delineation. We validate TASH by showing significant relationships between HG volumes obtained using manual labelling and using TASH, in three independent datasets acquired on different scanners and field strengths, and by showing good qualitative segmentation. We also present two applications of TASH, demonstrating replication and extension of previously published findings of relationships between HG volumes and (a) phonetic learning, and (b) musicianship. In sum, TASH effectively segments HG in a fully automated and reproducible manner, opening up a wide range of applications in the domains of expertise, disease, genetics and brain plasticity.

## Introduction

The human auditory cortex translates continuous, complex acoustic stimuli into signals allowing communication during speech perception, into musical experiences, and into cues allowing sound localization, to name a few. Heschl’s gyrus (HG), or the first transverse temporal gyrus, first described by Richard Heschl^1^, is an oblique convolution located on the inferior surface of the lateral fissure (also known as the Sylvian fissure), which runs transversely (mediolaterally) from near the insula towards the lateral part of the superior temporal gyrus^2^. HG includes the primary auditory cortex (PAC, or ‘auditory core’), this being the first cortical relay station of auditory information in the brain. Although the vast majority of non-invasive brain imaging studies on the auditory cortex have focused on auditory cortex functional recruitment^3–5^, the last 15 years have shown a growing number of structural magnetic resonance imaging (sMRI) studies on the anatomy (e.g. size, shape, thickness etc) of human auditory cortex, and of HG more specifically^6,7^. These studies have shown relationships between individual differences in HG size and morphology (i.e., gyrification) with individual differences in language skill, learning and expertise^8–15^, professional musicianship^16–20^ and reading disorders such as dyslexia^21–23^, to name a few. These auditory cortex structural differences could be due to individual differences in experience-dependent structural plasticity^24^, and/or to differences in pre-existing, possibly innate factors^25^. More generally, such macroscopic differences (i.e. ones visible with sMRI) are likely to be related to differences in underlying cellular and molecular differences^24^, which in turn likely partly account for the aforementioned relationships between gross anatomical differences and individual differences in behavioral skill, expertise and disease.

The reliable and reproducible localization and delineation of HG is necessary not only in studies on the anatomy of this brain region per se, but also in order to help in the localization of data obtained from other imaging modalities such as functional MRI (fMRI), positron emission tomography (PET) and diffusion tensor imaging (DTI)^26^. The anatomy of HG is highly variable across individuals and hemispheres, both in terms of size and morphology (i.e. of gyrification patterns)^1,27,28^. On average and in healthy populations, HG is larger in the left compared to the right hemisphere^27,29,30^. Furthermore, the most common gyrification patterns include single HG, common stem duplications (CSD, partial split of the gyrus by a sulcus intermedius) and full posterior duplications (FPD, two fully separated transverse temporal gyri, also known as complete posterior duplications)^6,31,32^, but in some cases a third or even additional gyri can be present^17^. Early cytoarchitectonic studies showed that the granular core comprising the PAC is located in the medial portion of HG^2,33^. However, later such work performed on larger samples of postmortem brains showed that although the medial aspect of HG consistently contains PAC, the extent to which PAC extends to adjacent transverse temporal gyri varies between hemispheres and brains^34^, and that the mapping between cytoarchitectonic borders and gyral/sulcal boundaries is poor^35^. In other words, HG is not synonymous with the PAC, but rather includes PAC.^2,33^. More recent, *in-vivo* myelin mapping studies have shown higher myelination values within the medial two thirds of HG, and higher myelination is thought to be one of the *in-vivo* markers of primary auditory cortex^36,37^. Although there is not a clear relationship between these gross anatomical features and cytoarchitecture, HG is macroscopically defined as comprising the most anterior transverse temporal gyrus, including common stem duplications when they are present, in particular when the intermediate sulcus spans less than half the length of HG^27^. Full posterior duplications, when present, are ascribed to the planum temporale (PT), which includes secondary auditory cortex^38^. Thus, using gross anatomical landmarks, the posterior border of HG (this being the anterior boundary of the PT) is typically defined as corresponding either to: a) the first complete sulcus posteriorly adjacent to HG (also known as Heschl’s sulcus (HS)), in the case of single HG, in the case of full posterior duplications or in the case of CSDs when the sulcus intermedius is short, or b) it is defined as corresponding to the sulcus intermedius in the case of common stem duplications where the sulcus intermedius is long^27,32^.

In this paper, we present a novel, automated toolbox serving to segment HG, here defined as the first transverse temporal gyrus together with existing CSDs but excluding FPDs, based on gross anatomical landmarks obtained from T1-weighted structural MRI data only. The fully automated nature of this toolbox allows to perform reproducible macroscopic feature selection in large datasets in which information from other imaging modalities (e.g. myelin mapping, tonotopy) is not available. Due to the large inter-hemispheric and inter-individual variability in the morphology of HG, the gold standard for the delineation of HG in structural MRI data is manual (i.e. visually guided) labelling^9,13,16,39^. This approach, however, is not fully reproducible and is thus error prone^40^, and is also very time consuming.

Automated approaches for the segmentation of specific, small brain structures based on structural MRI data (i.e. T1-weighted images) have been developed in the last years. These have mainly aimed to segment subcortical brain regions such as the caudate nucleus^41^, the thalamus^42^, the putamen^43^ and the hippocampus^44^. These approaches have included methods based on Bayesian models of shape and appearance^45^, probabilistic models^46^, fuzzy templates^47^, large deformation diffeomorphic metric mapping^48^ and decision fusion^49^. These automated methods have shown good performance for the segmentation of subcortical structures. This is likely facilitated by the fact that subcortical nuclei are constituted of closed surfaces, and thus, grey-white matter intensity contrast values can largely assist in improving segmentation performance. The automatic segmentation of structures such as HG, which lie on ‘open’ surfaces and are thus contiguous with adjacent structures, however, poses a much bigger challenge since the boundaries of such structures are more arbitrary and less easily identifiable using T1-weighted image properties such as grey/white matter contrast.

Only a few studies have attempted to develop methods to automatically segment HG specifically, using only gross landmarks from T1-weighted sMRI data^50–53^. For example, automatic delineation of the transverse temporal gyrus has been tackled by using sulci as landmarks. The approach, developed by Engel and colleagues^50^, segments HG using a deformable model of folding patterns that adapts to the curvature of the cortical surface. This approach uses anatomical gyral and sulcal landmarks to parcellate the auditory cortex, and then uses pattern recognition algorithms to label the auditory cortex. However, the high inter-subject variability of sulco–gyral landmarks poses problems for such pattern recognition algorithms, and also, this approach is not fully automated^50^. Another technique for the automated segmentation of HG has been proposed based on the construction of deformation-based probabilistic maps, but this approach is based on the use of a template, which biases results towards the anatomy of the selected template^53^. Yet another method relies on a partially automated algorithm for the anatomical parcellation of the auditory cortex, making use of a contextual pattern recognition method that relies on rendering a deformable prototype-based recognition method, and adding basic atlas information^52^. This approach, however, relies on training a machine-learning algorithm based on manually delineated surfaces, and as such, is not fully automated and still requires arbitrary decisions to be made regarding where to place regional boundaries. Therefore, there remains a need for fully automated algorithms for the segmentation and parcellation of the transverse temporal gyrus, especially in the context of studies where large sample sizes are involved.

One of the most widely used software packages allowing the fully automated segmentation of different cortical and subcortical brain regions is FreeSurfer^54^. This software performs parcellation of cortical and subcortical regions using probabilistic classification based on two different manually labeled atlases, these being the Destrieux^51^ and the Desikan atlases^55^. The former atlas, in particular, provides a more detailed parcellation of the auditory cortex than the latter, although it has not been tailored for the fine delineation of this brain region specifically. Indeed, it labels the transverse temporal gyrus (i.e. HG), the transverse temporal sulcus and the PT among a total of 74 cortical regions that are segmented in each hemisphere, to provide a complete labeling of sulci and gyri of the human cortex. Due to the high anatomical variability of the transverse temporal gyrus, and to the fact that Freesurfer has not been explicitly designed to segment this region specifically, the labelling of this brain region is prone to errors. For example, the most medial portion of HG is often erroneously excluded from the transverse temporal gyrus region of interest. Moreover, for the case of CSDs, parts or all of the second gyrus are sometimes assigned to the PT regardless of the length of the sulcus intermedius, even though according to conventional definitions of HG, the second gyrus ought to be assigned to HG in cases where the sulcus intermedius is short^27^. Conversely, again for the case of CSDs, parts of the second gyrus are sometimes erroneously assigned to the transverse temporal sulcus label. Freesurfer normally correctly assigns full posterior duplications (FPDs, i.e. second and third or other transverse temporal gyri) to the PT label.

Here, we present a novel method for the automated segmentation of HG. Our auditory cortex segmentation toolbox, called TASH (Toolbox for the Automatic Segmentation of HG), makes use of the output of the FreeSurfer structural segmentation pipeline, and then implements further steps to provide a finer segmentation of HG. By default, our pipeline segments the most anterior transverse temporal gyrus, including CSDs when present (i.e. regardless of the length of the sulcus intermedius), but excluding FPDs and further (i.e. 3^rd^ or 4^th^) transverse temporal gyri. We henceforth refer to this delineation as ‘HG’. Moreover, this pipeline is currently being adapted for alternative feature selection approaches (see Discussion). We first validate the method by testing for relationships between HG volumes obtained using manual labelling and using TASH, and by examining the quality of feature selection by TASH, in three independent sMRI datasets (the ‘musicianship’ data) obtained on different MRI scanners and at different magnetic field strengths. The purpose of validating our method across different datasets was not only to show that our validation replicates across different data, but also to show that TASH performs robust feature selection independently of scanner and field strength. Following the validation, we also provide two applications of our method to (a) this same, ‘musicianship’ data and also to (b) two additional sMRI datasets (the ‘phonetic learning’ data, also used for the toolbox development), to test for whether TASH replicates previously observed relationships between HG volume and (a) language skill, and (b) musicianship. Indeed, previous studies have shown, using manual labelling of HG, that people who are faster at learning to hear foreign speech sounds have a larger left HG volume compared to slower phonetic learners^9^, and also that both amateur and professional musicians have larger grey matter volumes of HG bilaterally compared to non-musicians^16,56^. We also tested for group differences in HG surface area and cortical thickness. We predicted that TASH, a fully automated method, would successfully replicate these previous manual labelling findings of (a) a larger left HG in faster compared to slower phonetic learners^9^, and of (b) bilaterally larger HG in professional and amateur musicians compared to non-musicians^16,56^, and that in the latter context TASH would extend these findings to new data.

## Methods

**Figure 1:**
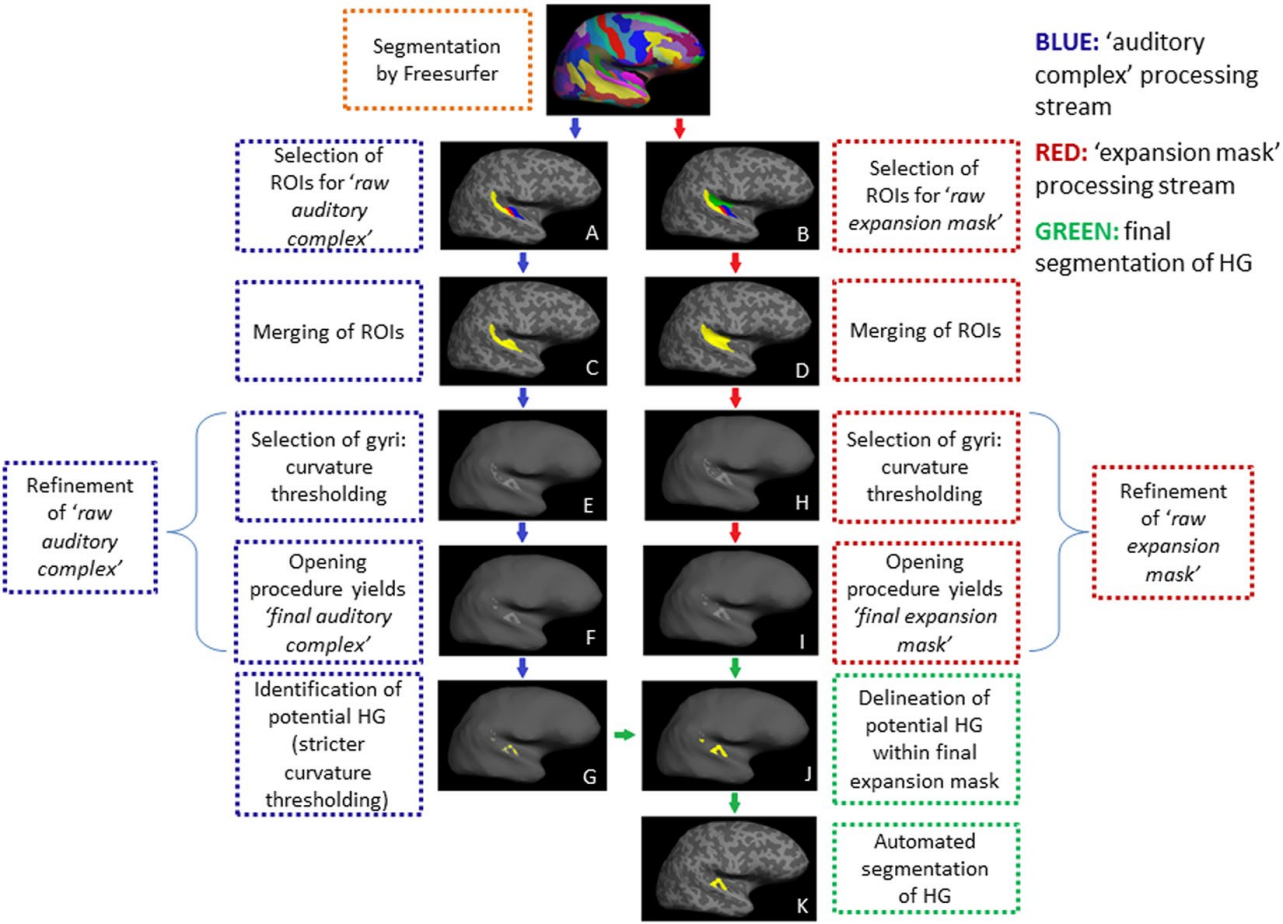
Schematic diagram illustrating the different steps of TASH.

TASH requires structural MRI images to first be processed by FreeSurfer. Further steps, as outlined schematically in Fig. 1, are then implemented on selected elements of the FreeSurfer output in order to yield fine HG segmentation. The toolbox environment consists of scripts written using the MATLAB R2012a software package^57^ (also tested on more recent versions of Matlab, e.g. Matlab 2018b) and TCSH Shell platform^58^, and also makes use of segments of code from FreeSurfer v5.3^54^.

For the development of this method we made use of two existing sMRI datasets^9,59^, henceforth called the ‘phonetic learning’ data. These included the fastest and slowest phonetic learners from two previous studies^9,59^ having examined the relationship between brain structure and phonetic learning skill, totaling 41 participants (71% females; mean age: 25.9 years). One of the T1 image datasets (scanning parameters reported in^9^) was acquired on a 1.5 T Signa Horizon Echospeed MRI scanner (General Electric Medical Systems, Milwaukee, WI), and the second T1 image dataset (scanning parameters reported in^59^) was obtained on a 1.5 T Philips Gyroscan scanner. Once developed, TASH was then validated in three independent sMRI datasets (i.e. not the same as those used for developing the method), henceforth called the ‘musicianship’ data. The details of the musicianship data are provided in Section 2.4, entitled ‘Validation and applications of the method’, and in Table 1. After the validation of TASH, we provide two applications of this toolbox to data in the contexts of (a) phonetic learning, using the data on which the method was developed^9,59^, and (b) musicianship, using the data on which TASH was validated (see Section 2.4). All of the data used for the development of TASH, for its validation and for the applications were acquired using procedures that were approved by the respective local research ethics committees. These included the Ethics Committee of the Medical Faculty of Heidelberg University, the Research Ethics Committee of the Montreal Neurological Institute and Hospital and the ‘Comité de protection des personnes Régional du Kremlin Bicêtre’. All subjects gave their informed consent for participation in the experiments.

**Table 1:** Data used for validation of TASH and for the musicianship application (see section 2.4.2.2): demographics (age and sex) of participants scanned on different MRI scanners. For the musicianship application, the data obtained on the Symphony 1.5 T and on the Trio 3.0 T scanners consisted of data in which HG volumes between musicians and non-musicians have previously been shown. The data obtained on the TrioTim 3 T scanner consisted of new data in which we aimed to extend previous findings of HG volume group differences.

Scanner	Group	Professionals		Amateurs		Non-musicians		All
	Sex	Fem	Male	Fem	Male	Fem	Male	Both
Symphony 1.5 T^16,39,56^	Number	5	5	6	4	1	2	23
	Age (mean)	28.2	24.4	29.0	56.2	53.0	58.5	36.2
	Age (std)	13.3	4.0	8.1	12.4	-	6.4	16.3
Trio 3.0 T^65,66^	Number	9	17	4	11	9	14	64
	Age (mean)	40.0	41.0	39.7	31.2	40.1	51.8	41.3
	Age (std)	8.2	9.1	8.7	13.5	15.8	8.0	12.2
Trio-Tim 3.0 T^67^	Number	14	19	24	26	5	6	94
	Age (mean)	39.1	39.1	29.4	31.2	26.0	29.7	33.2
	Age (std)	13.2	12.5	10.2	9.7	4.5	5.1	11.4
All fields	Number	28	41	34	41	15	22	181
	Age (mean)	37.5	38.1	30.6	33.7	36.3	46.4	36.4
	Age (std)	10.1	11.5	10.1	13.1	14.7	12.6	12.9

In the Methods section, we first provide an overview of TASH, followed by a description of the methods used for the validation and subsequently for the applications of this novel toolbox.

### Data acquisition

Individual subject T1 weighted structural MRI data are required as input to the TASH pipeline, with a recommended spatial resolution of 1.0 cubic millimeter. For good segmentation by FreeSurfer, it is recommended that voxels do not exceed 1.5 mm along any direction (see FreeSurfer user guidelines). Good gray/white matter contrast of the T1 images is crucial for accurate results.

### Initial processing by FreeSurfer

Depending on the quality of the T1 images, if noise is identified, denoising is a recommended step that can improve segmentation and labelling by FreeSurfer. For denoising, we recommend use of the spatially adaptive non-local means (SANLM) filter^60^ (University of Jena, Germany), available as a MATLAB based toolbox in VBM 8 (within SPM 8) and in CAT 12 (within SPM 12)^61^. This filter removes both equally distributed and/or locally specific noise while preserving edges, which is the key information required for good grey/white matter segmentation and for subsequent cortical surface reconstruction. SANLM can also be combined with the ‘patch and pixel similarity’ approach for denoising^62^. Following denoising (if required), the native space T1 data are processed by FreeSurfer’s brain structural pipeline^63^ to achieve grey/white matter segmentation, cortical reconstruction and parcellation of different brain regions.

### TASH pipeline

#### Selection and merging of auditory cortex ROIs

TASH makes use of specific labels, or regions of interest (ROIs), in and around the auditory cortex arising from Freesurfer’s Destrieux atlas parcellation^51^. The initial selection of a broader region around HG allows for the subsequent refinement of auditory cortex feature selection while accounting for the large variability in the anatomy of this region across individuals. The following cortical regions are extracted from each subject’s FreeSurfer’s segmentation using TCSH shell scripts (see panels A and B in Fig. 1): (1) HG, called the ‘transverse temporal gyrus’ in FreeSurfer’s Destrieux atlas, (2) HS, called the ‘transverse temporal sulcus’, (3) the PT and (4) the posterior Sylvian fissure, called the ‘posterior segment of the lateral sulcus’. The first 3 ROIs are then merged to form a first ‘raw auditory complex’ (panel C in Fig. 1), and all 4 above ROIs are merged to form the ‘raw expansion mask’ (panel D in Fig. 1). As described below, the ‘auditory complex’ is subsequently used as a search region for locating the crown of HG (see the blue arrow stream in Fig. 1), while the ‘expansion mask’ is used to delineate (i.e. to outline) the full extent of the identified gyrus/gyri from those (see red arrow stream in Fig. 1).

#### Refinement of the raw auditory complex (blue arrow stream in Fig. 1)

In order to refine the raw auditory complex (i.e. the merged HG, HS & PT region), first, we identify all of the gyri within this region by applying a threshold to the curvature values determined by Freesurfer on the gray-white matter boundary surface, thus selecting only the corresponding vertices with negative curvature values. Indeed, within Freesurfer, vertices with negative curvature values are locally convex and thus correspond to points on gyri, and conversely, ones with positive curvature values are locally concave, and thus correspond to points on sulci. We thus first apply a threshold of 0 to the mean curvature values in order to retain only vertices having negative curvature values (panel E in Fig. 1). Once the vertices corresponding to gyri are selected, these are submitted to an opening procedure (i.e. erosion followed by dilation)^64^ (panel F). This serves to eliminate regions that are erroneously included within the FreeSurfer ROIs which contribute to this mask, and which can result in the erroneous selection of gyri which are not HG but which lie further posteriorly along the PT, including thin formations that run along the superior temporal gyrus but that do not belong to HG. The opening procedure uses a disk having a radius equal to 2 times the edge length on the vertex space. It serves to first remove three layers of vertices at the edge of the selected patch (i.e. the three external-most layers) within the 2-D graph representation, thus discarding thin formations having a width of up to six vertices, and subsequently to restore the eroded outer-most layers of HG, but this time no longer including the thin formations. This step yields the ‘final auditory complex’ (panel F in Fig. 1), and includes all transverse temporal gyri that are located within the HG/PT region.

#### Identification of gyral crown (blue arrow stream in Fig. 1)

Within the ‘final auditory complex’, the crowns (i.e., parts of highest curvature) of the existing gyri are identified by applying a threshold of −0.1 to the mean curvature values of the corresponding points on the gray-white matter boundary surface, thus retaining only the vertices with a mean curvature lower than −0.1 (panel G in Fig. 1). Vertices that survive this thresholding procedure correspond to the crowns of the gyri within the HG/PT complex, on which an expansion procedure is subsequently applied (see section 2.3.5) in order to recover the full extent of the gyri within a larger expansion mask (see next section).

#### Refinement of the expansion mask (red arrow stream in Fig. 1)

This step serves to refine the mask within which the gyri identified in the above step are allowed to ‘grow back’. As described in section 2.3.1, the raw expansion mask includes HG, HS and PT but also the posterior segment of the lateral fissure, located medially to the auditory complex (panel D). Inclusion of this latter region in the expansion mask serves to ensure that TASH correctly selects the medial-most portion of HG, which is often erroneously excluded in the FreeSurfer transverse temporal gyrus label (see Introduction). The raw expansion mask is refined (see red arrow stream in Fig. 1) following similar procedures as were used for refining the raw auditory complex. First, in order to restrict the final expansion mask to gyri, we apply a threshold of 0 to the mean curvature values in order to retain only vertices having negative curvature values (panel H). This gyrus-specific mask is then further refined using an opening procedure, again using a disk having a radius equal to 2 times the edge length on the vertex space. This provides the ‘final expansion mask’ (panel I).

#### Delineation of full extent of auditory cortex gyri

The gyral ‘crowns’ identified as described in Section 2.3.3 are then ‘grown back’, or expanded within the final expansion mask, in order to recover the full extent of each gyrus. This is done by selecting all adjacent vertices having mean curvature values below 0 within the limits of the expansion mask (panel J and green arrows in Fig. 1).

#### Identification of HG

The location of the volumetric center of each gyrus delineated in the above step (panel J) is then determined. An automatic check for additional remaining clusters is then performed, and when found, smaller clusters than 100 vertices are eliminated. Next, to identify the gyrus that is deemed to correspond to HG (i.e. the most anterior transverse temporal gyrus, including CSDs), we select the most anterior of the identified gyri, based on their volumetric center (panel K). This is the final step for the delineation of HG, which is saved in FreeSurfer’s ‘label’ file format.

#### Visualization of output

The HG segmentation (i.e. in the ‘label’ file format), can be viewed using FreeSurfer’s visualization tools (e.g. freeview, tksurfer), where it can be overlaid onto the participant’s brain structural scan. In order to also be able to open the images on other platforms, the HG segmentations are additionally converted from the ‘label’ to the ‘tiff’ file format.

#### Quantification of results

Scripts adapted from FreeSurfer are then used to extract measures including the volume, surface area, mean thickness and its standard deviation, mean curvature, Gaussian curvature, curvature index and folding index of the HG segmentations. For extracting the above measures, the ‘mri_anatomical_stats’ command is used, which allows to extract grey (and not white) matter volumes. These results can then be analyzed across subjects using standard parametric statistics, non-parametric statistics, machine learning, or other approaches, depending on the goals and questions of the study. Note that the above measures are in native space, and that when performing group statistics, measures such as mean hemispheric (or cortical volume, surface area, thickness, etc) need to be controlled for statistically.

### Validation and applications of the method

#### Validation

We validated our method by testing to see if left and right HG volumes obtained using TASH correlate well with those obtained using manual labeling, in three independent datasets, this being the ‘musicianship’ data. For comparison purposes, we also compared the manually labelled HG volumes to those obtained using FreeSurfer’s Destrieux parcellation of the transverse temporal gyrus^51^, in these same datasets. For the present validation, we used three sets of previously acquired T1-weighted sMRI data (N = 181 in total), characteristics of which are summarized in Table 1. We included subsets of data from previously published studies on differences in HG volume between professional and amateur musicians and non-musicians^39,65^, as well as data from studies on auditory skill^66^ and on the structure and function of the auditory cortex^17,67^. More specifically, we included all the healthy non-musician and (amateur and professional) musician participants from the 5 above studies who were healthy 18–65 years old adults (with the exception of two 17-year old individuals) having been scanned using the same protocol (see below) and 12 channel head coil. None had any previous neurological or auditory disorders. As can be seen in Table 1, we included data from 23 participants scanned on a Symphony 1.5 T scanner, 64 participants scanned on a Trio 3.0 T scanner and 94 participants scanned on a Trio-Tim 3.0 T scanner. All of these data were acquired using 12-channel head coils and a standardized scanning protocol (MPRAGE, 176 contiguous sagittal slices, 1 mm^3^ isotropic resolution, TR 1930 ms, TE 4.38 ms). These data included sMRI scans of 69 professional musicians, 75 amateur musicians and 37 non-musicians. There was a similar proportion of males and females across groups (F = 0.201, p = 0.818), but the groups differed significantly with respect to mean age (F = 8.893, p < 0.001), with the amateurs being significantly younger than both professionals (t = 2.84, p = 0.005) and non-musicians (t=3.93, p < 0.001).

Manual segmentation of bilateral HG on pseudo-anonymized data was performed by one of the authors of this paper who is highly experienced with the manual labeling of HG (PS), and who has published a number of papers regarding the anatomy of HG in musicians and in other populations^16,17,39,65–67^. Labelling involved use of a stepwise semi-automated procedure in native space, using BrainVoyager QX 2.8 software^68^. The preprocessing of T1-weighted images included inhomogeneity correction and AC-PC alignment. Histograms of MR intensities were generated, and only voxels thought to correspond to grey matter were included. Individual HG were segmented slice-by-slice, based on sagittal images. HG was defined as including the medial two thirds of the most anterior transverse temporal gyrus within the supratemporal plane, delimited anteriorly by the first transverse temporal sulcus and posteriorly by HS. Note however that the anterior border of the medial two thirds according to this manual labeling definition was in fact often in close proximity to the anterior border as determined by Freesurfer and TASH, because the manual labelling definition of the anterior end of the *full* extent of HG was considerably more liberal (i.e. antero-lateral) than is considered the case within Freesurfer and within other manual labeling studies^9,10,27^. In particular, the anterior border of the full extent of HG in the manual labeling approach considered by the rater in the current work (PS and JB) was defined as extending until an imaginary line crossing the anterior commissure^17,23,39,66,69,70^. Hence, as can be seen in the left and right HG of the prototypical case shown in Supplementary Fig. 1, the antero-lateral border of HG was in fact often similar when comparing the manual labels to the TASH and Freesurfer ones. For the manual labeling, in the case of HG duplications (e.g. CSD or FPD), the border between HG and PT was defined as the most anterior complete transverse HS, consistent with the definition used by TASH. The medial boundary of HG was identified using a drawn line connecting the medial end of FTS to the medial end of HS, and the lateral boundary was determined using a fixed distance of 24 mm spanning laterally along the direction of HG, with the average length of HG being 36 mm. Manual labeling was blind to group, and was performed independently of the TASH pipeline, and vice versa. For evaluating the internal consistency of our semi-automatic manual labeling procedure, we assessed inter-rater reliability analyses on a randomly selected subsample of 52 participants, on which HG volumes were calculated by three different raters (PS and two new raters). Inter-rater reliability, assessed by Pearson’s correlations, were very high for both the right (r (52) = 0.99, 0.99 and 0.99) and the left hemispheres (r (52) = 0.97, 0.97 and 0.98).

Note that there are two differences in the way that TASH extracts HG volumes compared to the way this is done during manual labeling. First, when manual labelling is performed, the gyri are selected from their upper convolution down to the bottom of the sulci that adjacently delimit them, whereas when gyri are selected within FreeSurfer and TASH, the selection does not go all the way down to the sulcus but stops approximately mid-way between the bottom of the sulcus and the upper-most point of the gyrus. This is because of the way that FreeSurfer (and thus TASH) defines gyri; gyri are defined as corresponding to vertices having negative curvature values. Second, during manual labelling, as mentioned above, the medial two-thirds of HG was selected, while in FreeSurfer and TASH, the entire gyrus was selected. As described above, however, despite this, due to the more liberal definition of the anterior border of the full extent of HG during manual labeling, in many cases the antero-lateral border corresponding to the medial two-thirds during manual labeling was actually similar to the lateral border of HG as considered within Freesurfer, and thus also within TASH (see Supplementary Fig. 1). Due to the above differences between these approaches, the correlations between manually labelled HG volumes and those obtained using TASH and FreeSurfer thus somewhat underestimate the strength of existing relationships, whereas the strength of the relationship between HG volumes obtained using FreeSurfer and TASH should not be affected. Note also that in all three methods, the grey matter volumes (surface areas, etc.) generated are in native space, making it unnecessary to control for cortical volumes when evaluating differences among these 3 labeling methods.

#### Applications

Following the validation of TASH, we applied this toolbox to the ‘phonetic learning’ and to the ‘musicianship’ data that were used to develop and to validate TASH, respectively, with the goal of testing for whether HG volumes obtained automatically using TASH would replicate and extend previously published demonstrations of larger HG volumes in faster phonetic learners and in musicians, respectively.

##### Phonetic learning application

We used TASH to obtain left HG volumes in the brain structural data of participants in whom we have previously shown, using manual labelling and VBM, positive relationships between left HG volumes and phonetic learning scores (i.e. the ‘phonetic learning’ data). As previously noted, this subset of the data was used to develop the TASH toolbox, however, none of these data were used for the validation of TASH (described in section 2.4.1). For this first, phonetic learning application of TASH, we included data from the fastest and slowest phonetic learners previously described in Golestani and colleagues 2002 and 2007^9,59^, totaling 41 participants (71% females; mean age: 25.9 years). In these previous studies, larger numbers of participants (N = 65^9^; N = 59^59^) had been behaviorally trained to learn the ‘difficult’ Hindi dental-retroflex phonetic contrast, and the fastest and slowest phonetic learners had been selected for MRI scanning. Here, we aimed to replicate the previous findings of larger left HG in faster compared to slower learners, using TASH.

##### Musicianship application

For the second, musicianship application of TASH, we used the left and right HG volumes obtained using TASH in the three ‘musicianship’ sMRI datasets, combined across the different scanners/field strengths. As previously noted, this data was also used for the validation of this method (see descriptions in the ‘Validation’ section 2.4.1 and in Table 1). Part of these data has previously been used to show, using manual labelling of HG, that musicians, whether professional or amateur, have larger HG volumes bilaterally than non-musicians^16,39,56,65,66^; here we aimed to replicate these findings^16,39,56,65,66^ and extend them^67^ to a larger dataset, using HG volumes obtained automatically with TASH.

### Ethical approval

All procedures performed in the studies were in accordance with the ethical standards of the institutional and/or national research committee and with the 1964 Helsinki declaration and its later amendments or comparable ethical standards. The respective ethics committees included: the Ethics Committee of the Medical Faculty of Heidelberg University, the Research Ethics Committee of the Montreal Neurological Institute and Hospital and the ‘Comité de protection des personnes Régional du Kremlin Bicêtre’.

## Results

### Validation of the method

**Figure 2:**
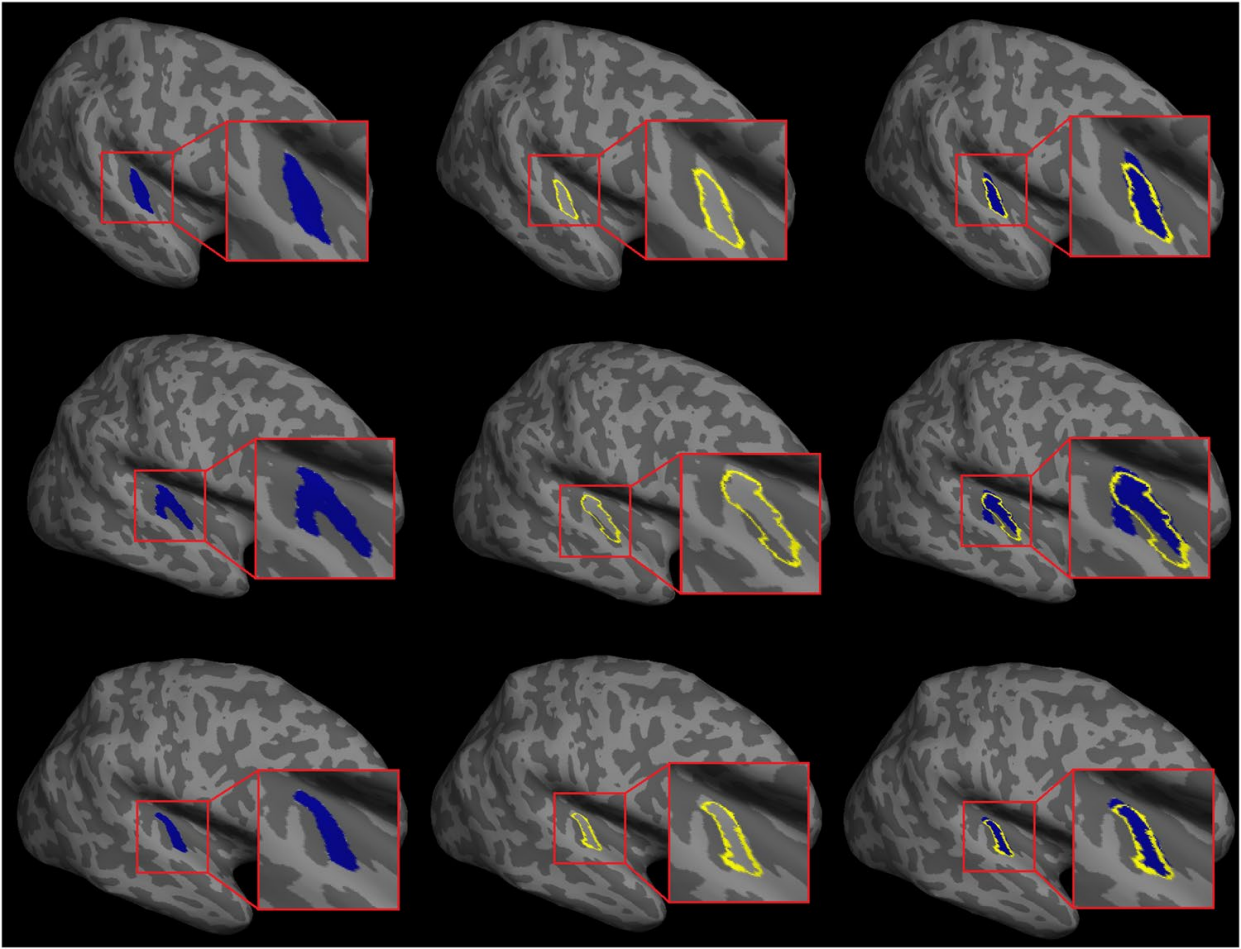
Examples of TASH (left, in blue) and FreeSurfer (middle, yellow outlines) HG segmentations in the right hemispheres of three representative participants, and the overlays of these (right). Light grey regions depict gyri and dark grey ones depict sulci. Red squares on the right of each panel show zoomed-in views of the auditory cortex. Top: single transverse temporal gyrus (HG), middle: common stem duplication (CSD), bottom: full posterior duplication (FPD).

First, we examined the convergence in HG volumes obtained using FreeSurfer and TASH. For this, we combined the three musicianship datasets (i.e. those in musicians, amateurs and controls), and computed the correlations between HG volumes obtained using the two methods, for the left and right HG separately. Results showed correlations of r(180) = 0.73 (p < 0.001) for left HG, and of r(180) = 0.54 (p < 0.001) for right HG. The relatively weaker correlation in the right hemisphere suggests that TASH and FreeSurfer differ more in terms of how they segment HG in the right compared to the left hemisphere (see Discussion for possible explanations for this). Qualitatively, we observed that as expected, TASH provides relatively finer feature selection than FreeSurfer in that some of the errors that are regularly committed in the FreeSurfer labelling are corrected by TASH. Figure 2 provides examples of the TASH and the FreeSurfer segmentations of the right HG in three representative participants: one with a single transverse temporal gyrus (top row), one with a common-stem duplication (middle row) and one with a full posterior duplication (bottom row). It can be seen in these examples that the FreeSurfer transverse temporal gyrus ROIs (see yellow outlines, in the middle column) erroneously exclude the most medial part of this gyrus. Also, in particular in the case of common stem duplications (middle row), the FreeSurfer transverse temporal gyrus label ‘spills over’ into the adjacent sulcus (dark grey zone), and also it selects part but not all of the second transverse temporal gyrus. A visual comparison among manual, TASH and FreeSurfer segmentation results for two representative participants is available in Supplementary Fig. S1.

Next, in order to quantitatively validate TASH, we compared the left and right HG volumes obtained using manual labelling to those obtained using TASH and also to those obtained using FreeSurfer. We did this in the data acquired on the three different scanners (and thus at both field strengths) and in the left and right hemispheres separately, in order to explore the relative strength of the relationships in these different data. Bivariate correlations revealed intermediate to strong relationships, ranging from r = 0.65 to r = 0.89, between manually labelled HG volumes and TASH volumes (Table 2). All of these correlations were significantly stronger than those between manually labelled volumes and ones obtained using FreeSurfer, as revealed by Fisher’s r-to-z transformations. Table 2 provides an overview of the correlations, and of significance testing.

**Table 2:** Pearson’s correlations between HG volumes obtained using manual labelling versus TASH and versus Freesurfer. Fisher’s r-to-z transformation (1-tailed) was used to assess differences between correlations; all results were significant at α = 0.05.

Scanner	Hemisphere	Correlation with manual labelling		Fisher’s r-to-z transformation	
		TASH	FreeSurfer	z-score	p-value
Symphony 1.5 T	Left	0.72	0.53	1.85	0.032
	Right	0.89	0.57	3.35	<0.001
Trio 3.0 T	Left	0.79	0.65	2.56	0.005
	Right	0.73	0.42	3.09	0.001
Trio-Tim 3.0 T	Left	0.65	0.50	2.31	0.010
	Right	0.69	0.38	4.34	<0.001
All	Left	0.72	0.55	4.30	<0.001
	Right	0.72	0.39	6.16	<0.001

### Applications of the method

#### Phonetic learning application

As a first application of our method, we tested to see if HG volumes obtained using TASH correlate with phonetic learning scores, as has previously been shown using manual labeling and voxel-based morphometry (VBM) in these same participants (see Methods). Partial correlations were performed between left HG grey matter volumes obtained using different methods (manual labeling, TASH and FreeSurfer) and phonetic learning scores, controlling for mean left hemisphere cortical volumes. Results revealed that the volumes obtained using TASH correlate positively with phonetic learning scores [r(38) = 0.45, p = 0.001, 1-tailed], replicating previous findings^29^. The partial correlation between left HG volumes obtained using manual labelling and phonetic learning was also significant, and of similar strength [r(38) = 0.44, p = 0.002, 1-tailed]. The relationship between left HG volumes obtained using FreeSurfer and phonetic learning was, however, qualitatively relatively weaker [r(38) = 0.29, p = 0.033, 1-tailed]. We also found a significant relationship, using partial correlations, between phonetic learning scores and left HG surface area obtained using TASH [r(38) = 0.38, p = 0.007, 1-tailed], but not between phonetic learning and cortical thickness [r(38) = 0.06, p = 0.365, 1-tailed].

#### Musicianship application

**Figure 3:**
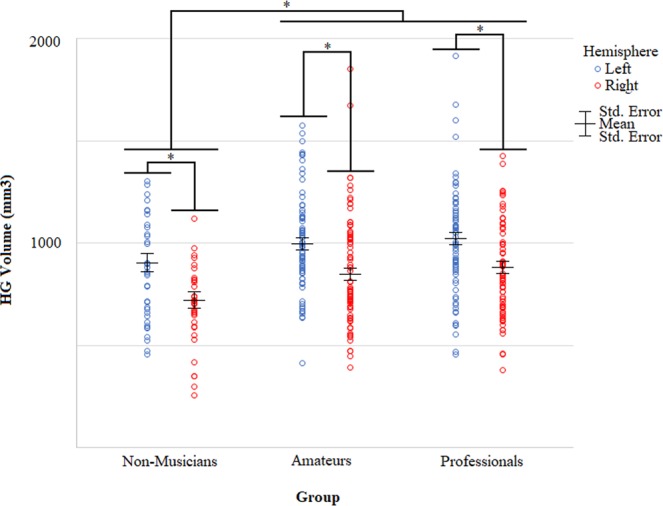
Average HG grey matter volumes for professionals, amateurs and non-musicians in the left (blue) and right (red) hemispheres. *Significant differences at α = 0.05 (2-tailed).

In the second TASH application, we tested for group differences in HG grey matter volumes obtained using TASH in the musician and non-musician participants (N = 181) that the method was validated on (see Section 3.1). We analyzed the volumes using a mixed ANCOVA, with group (non-musicians, amateurs and professional musicians) as the between-subjects factor and with hemisphere as the within-subjects factor, controlling for the covariates of age, sex, whole brain cortical volume and scanner. There was only a significant effect of group [F(2,174) = 6.84, p = 0.001]; the effect of hemisphere [F(1,174) = 0.19, p = 0.668] and the group-by-hemisphere interaction [F(2,174) = 0.17, p = 0.845] were not significant. Post-hoc pair‐wise comparisons (Bonferroni corrected) on bilateral HG volumes (i.e. left and right hemisphere combined) controlling for age, sex, whole brain cortical volume and scanner showed that professional and amateur musicians have significantly larger HG volumes bilaterally compared to non-musicians (see Table 3 and Fig. 3 for HG volumes, Table 4 for pairwise comparisons). Furthermore, based on previous research showing a normative left > right asymmetry in HG volumes^27^, we also tested for an overall asymmetry in HG volumes (i.e. across all the participants). A paired t-test over all groups combined revealed, as expected, a significantly larger grey matter volume in the left compared to right HG [t(180) = 7.135, p  < 0.001].

**Table 3:** Left and right HG grey matter volumes in professional musicians, amateurs and non-musicians. *Adjusted for the covariates of age, sex, cortical volume and scanner.

Category	Hemisphere	HG Volume* (mm^3^)		95% Confidence Interval	
		Average	Std. Error	Lower Bound	Upper Bound
Non-musicians	Left	898	41	817	978
	Right	715	39	639	791
Amateurs	Left	990	29	933	1047
	Right	840	28	786	895
Professionals	Left	1015	30	956	1073
	Right	874	28	818	929

**Table 4:** Post-hoc pairwise comparisons: paired t-tests (2-tailed) assessing differences in bilateral HG grey matter volume between professional musicians, amateurs and non-musicians, adjusted for age, sex, whole brain cortical volume and scanner. Comparisons are corrected for multiple comparisons (Bonferroni). *Indicates significance at α = 0.05.

Pairwise Comparisons	Mean difference	Std. Error	t-value	p-value
Professionals Vs Amateurs	29.2 mm^3^	32.5 mm^3^	0.898	1.000
Professionals Vs Non-Musicians	138.4 mm^3^	38.7 mm^3^	3.576	0.001*
Amateurs Vs Non-Musicians	109.2 mm^3^	39.7 mm^3^	2.751	0.020*

Additionally, we tested for group differences in the HG surface area and cortical thickness measures obtained using TASH in these musician and non-musician participants (N = 181). We analyzed surface area using a mixed ANCOVA, with group (non-musicians, amateurs and professional musicians) as the between-subjects factor and with hemisphere as the within-subjects factor, controlling for the covariates of age, sex, whole brain cortical area and scanner. Like for HG volume, for surface area also there was only a significant effect of group [F(2,174) = 4.3, p = 0.014]; the effect of hemisphere [F(1,174) = 0.54, p = 0.464] and the group-by-hemisphere interaction [F(2,174) = 1.14, p = 0.321] were not significant. Post-hoc pair‐wise comparisons (Bonferroni corrected) on bilateral HG surface area (i.e. left and right hemisphere combined) controlling for age, sex, whole brain surface area and scanner showed that professional musicians have significantly larger HG surface areas bilaterally compared to non-musicians (see Table 5 for pairwise comparisons). Furthermore, we also tested for an overall asymmetry in HG surface area (i.e. across all the participants). A paired t-test over all groups combined revealed a significantly larger surface area in the left compared to the right HG [t(180) = 6.832, p < 0.001].

**Table 5:** Post-hoc pairwise comparisons: paired t-tests (2-tailed) assessing differences in bilateral HG surface area between professional musicians (μ = 310 mm^2^), amateurs (μ = 297 mm^2^) and non-musicians (μ = 275 mm^2^), adjusted for age, sex, whole brain surface area and scanner. Comparisons are corrected for multiple comparisons (Bonferroni). *Indicates significance at α = 0.05.

Pairwise Comparisons	Mean difference	Std. Error	t-value	p-value
Professionals Vs Amateurs	12.4 mm^2^	09.8 mm^2^	1.265	0.209
Professionals Vs Non-Musicians	35.0 mm^2^	11.9 mm^2^	2.941	0.004*
Amateurs Vs Non-Musicians	22.6 mm^2^	12.1 mm^2^	1.868	0.064

We also analyzed HG cortical thickness measures using a mixed ANCOVA, with group (non-musicians, amateurs and professional musicians) as the between-subjects factor and with hemisphere as the within-subjects factor, controlling for the covariates of age, sex, whole brain mean thickness and scanner. Again, there was only a significant effect of group [F(2,174) = 3.25, p = 0.041]; the effect of hemisphere [F(1,174) = 0.706, p = 0.402] and the group-by-hemisphere interaction [F(2,174) = 0.486, p = 0.487] were not significant. Post-hoc pair‐wise comparisons (Bonferroni corrected) on bilateral HG cortical thickness (i.e. left and right hemisphere combined) controlling for age, sex, whole brain mean thickness and scanner showed that professional and amateur musicians have significantly higher HG cortical thickness bilaterally compared to non-musicians (see Table 6 for pairwise comparisons). Furthermore, we also tested for an overall asymmetry in the mean HG cortical thickness (i.e. across all the participants). A paired t-test over all groups combined did not reveal hemispheric differences [t(180) = 0.384, p = 0.701].

**Table 6:** Post-hoc pairwise comparisons: paired t-tests (2-tailed) assessing differences in bilateral HG cortical thickness values between professional musicians (μ = 2.40 mm), amateurs (μ = 2.41 mm) and non-musicians (μ = 2.32 mm), adjusted for age, sex, whole brain cortical volume and scanner. Comparisons are corrected for multiple comparisons (Bonferroni). *Indicates significance at α = 0.05.

Pairwise Comparisons	Mean difference	Std. Error	t-value	p-value
Professionals Vs Amateurs	0.015 mm	0.030 mm	0.500	0.630
Professionals Vs Non-Musicians	0.078 mm	0.037 mm	2.108	0.035*
Amateurs Vs Non-Musicians	0.092 mm	0.037 mm	2.486	0.015*

## Discussion

In this paper, we present the TASH toolbox, a novel, automated method for the segmentation of HG. This toolbox makes use of and builds upon the FreeSurfer pipeline in order to perform fine segmentation of the first transverse temporal gyrus (i.e. HG), including existing CSDs. We validated this segmentation method by showing that HG grey matter volumes obtained using TASH correlate well with those obtained using manual labelling in three independent structural MRI datasets (N = 181 participants in total), acquired on three different scanners at different field strengths. Thus, we show replication in our validation of TASH, demonstrating that it is robust to differences in scanner and field strength. Manual delineation for the labeling of cortical regions is still the gold standard for the fine segmentation of the auditory cortex, despite its drawbacks regarding reproducibility. Indeed, manual labelling of cortical regions is not only monotonous and time consuming, but in addition, the high anatomical variability of small structures such as HG and its existing duplications results in labelling errors and lack of reproducibility^40,52,71^. Given that our method is automated, it allows for fully reproducible feature selection, and is not subject to arbitrary and subjective decisions regarding the location of a boundary or regarding the presence of a gyrus, based, for example, on its apparent height as seen on a particular view of the brain. TASH provides improved segmentation of HG compared to FreeSurfer, both qualitatively and quantitatively. In particular, certain systematic errors arising from the FreeSurfer pipeline in the labelling of such a small and variable structure as HG are rectified by our pipeline. TASH can thus be used as a complementary tool to Freesurfer, in studies where there is specific interest in the detailed and accurate segmentation of HG.

We find that TASH and FreeSurfer converge less in terms of how they segment HG in the right compared to the left hemisphere. This might be due to several reasons, one being that the most anterior HG tends to be shallower in the right compared to the left hemisphere^65,72,73^, possibly making it more difficult for a segmentation tool that is not fine-tuned for such a small and variable structure as HG to accurately segment the region. Another reason for the relatively weaker convergence between right HG segmentations with TASH vs with FreeSurfer may be related to the fact that there tends to be greater morphological variability in the STG in the right compared to the left hemisphere in healthy populations^27,74^. Our validation dataset included data from healthy controls and also from musicians; it has been shown that also in musicians, the gyrification index tends to be higher in the right compared to the left HG, even when only considering CSD morphotypes^17^. Thus, again, it’s possible that TASH performs finer feature selection in the right HG than does FreeSurfer by virtue of TASH being explicitly designed to perform fine HG segmentation that is robust to variations in the anatomy of this region.

We also applied TASH to two datasets which have previously been used to show relationships, using manual labelling, between HG volumes and (a) phonetic learning^9^ and (b) musicianship status^16,56^. In the first application of our method, we showed that, as expected, left HG volumes obtained using TASH correlate positively with phonetic learning skill, replicating previous findings^9^. We also extended these findings to show a significant positive relationship between phonetic learning and left HG surface area. In the second application, TASH revealed larger HG volumes bilaterally in professional and amateur musicians compared to non-musicians, again replicating previous findings^16,56^ and extending them to a larger dataset. TASH also revealed group differences in HG surface area and cortical thickness; for surface area, only the professionals had higher values than non-musicians, and for thickness both professionals and amateurs had higher values than non-musicians. In one of the original manual labeling studies on this topic, it had been shown that professional and amateur musicians have larger HG volumes in the medial two-thirds of the first transverse temporal gyrus (with the border of the full extent of the transverse temporal gyrus being relatively liberally defined)^16^, however, in a later such study these volume differences were shown for both the medial and also for the lateral HG^56^. A larger volume of a gyrus (or of gyri, in the case of common stem duplications) is associated with a relatively larger surface area, as also shown in our results, and thus also with a relatively greater overall presence of superficial cortical layers compared to what can be expected to exist in smaller gyri. Interestingly, it has recently been shown that within the primary auditory cortex (i.e. in regions likely to be comprised by HG), there is greater processing complexity in superficial cortical layers^75^. Thus, a speculative explanation for larger bilateral HG in musicians and of a larger left HG in faster phonetic learners is that there may be a relatively greater presence of superficial layers in the HG of these populations, and this might underlie a capacity for better processing of complex or subtle musical and linguistic sounds these groups. It remains to be established how differences in the relative proportion of different cortical layers in gyri versus sulci^76^ might relate to overall gyrification patterns and to macroscopic differences in volume or surface area observed across people with different degrees of musical or linguistic skill. These questions can be explored in future, laminar resolution functional and structural imaging studies not only of cases with single transverse temporal gyri or with common stem duplications but also of more complex gyrification patterns.

There were a few differences in terms of how HG was defined during manual labeling versus within TASH. As described above (see Methods), in the current study the manual labeling considered the medial two-thirds of HG (i.e. ‘medial HG’), whereas TASH considers the full extent of HG. Despite this apparent difference across methods, the way that the lateral border of medial HG was defined during manual labeling actually corresponded relatively well to the lateral border of HG as determined by TASH (this was due to differences in how the full extent of HG is defined across methods, see the Methods section). Given, however, that during manual labeling the lateral border of the medial two-thirds of HG was defined using a fixed distance spanning laterally along the direction of HG, we expect that the correlations that we report between HG volumes obtained using TASH compared to manual labelling actually underestimate the strength of the true relationship between the two (i.e. the manually obtained volumes are likely more variable than they would have been had a ratio of the distance rather than an absolute distance been used for determining the medial two-thirds of HG). Also, there was a difference between the way in which the current analyses were implemented for examining the relationship between HG volumes and language/musical skill compared to those reported in the original studies: in the original studies, brain structural scans were normalized before HG were manually labelled, whereas in the current study, given that TASH generates grey matter volumes in native space, hemispheric cortical volumes were controlled for statistically (note however that in order to allow better comparability with TASH and FreeSurfer, the manual labeling performed for the validation (and applications) in the current study was done in native space).

Our replications of previous findings of larger HG volumes in relation to language learning and to musicianship using our fully automated approach further validate our method in showing that it can be used to uncover brain structure–behaviour relationships in a fully automated and replicable manner. This opens doors to numerous applications for studies where fine assessment of HG volumes (or surface area, thickness, etc) is needed, and in particular in studies where large sample sizes are involved. Further, TASH is a surface-based approach which makes use of T1-weighted structural MRI data. As such, it relies only on macroscopic structural landmarks for auditory cortex feature selection. Thus, it is complementary to functional imaging studies in terms of helping to localize functional responses in and around HG^26^, and also to DTI studies, where it can for example be used to determine seeds for tracking of auditory cortex white matter fibers^77^.

The version of our pipeline that has been described in this paper is designed to include CSDs but to exclude FPDs. We are currently also extending TASH to allow a more flexible approach to feature selection, one that can be decided upon by researchers based on their research goals and hypotheses. For example, previous work has shown associations between duplications (and triplications, etc) of the transverse temporal gyrus and musical/language skills^8,14,17^. Thus, there is a need for automated and reliable methods to allow the segmentation not only of HG but also of fully duplicated second (and third, etc., when present) transverse temporal gyri (e.g. FPDs), in individual subjects. Thus, future developments of TASH will include versions of the pipeline which will allow the selection of: (a) both CSDs and FPDs; (b) only the most anterior gyrus, even in the case of CSDs; (c) the medial two-thirds of the most anterior gyrus, region which corresponds most closely to the auditory core^2,33,37^; (d) HG plus anterior secondary auditory regions (i.e. the planum polare); (e) HG plus posterior secondary auditory regions (i.e., including possible duplications, triplications etc); and f) all transverse temporal gyri plus anterior and posterior auditory regions together. Thus, more generally, different implementations of TASH can allow to run several different feature selection versions on the same data, allowing comparison of HG volumes or of other measures (i.e., surface area, thickness, other) based on the selection of different auditory subregions or combinations thereof. Studies involving manual labelling have also done this^16,22^, but at the cost of many hours of work, and of not fully reproducible segmentations. It should be noted that the HG volumes and other measures (e.g. surface area, thickness) extracted using TASH can be assessed not only using conventional statistics, but also using other methods, such as machine learning. If multiple dependent measures such as HG volume, surface area, thickness, and curvature are extracted and assessed conjointly, then machine learning can serve to pinpoint which of those anatomical measures most clearly differentiate individuals with respect to the effects of interest (i.e., basic auditory processing, music or language expertise, disease or other).

Normative studies (i.e., in healthy controls) have revealed a hemispheric asymmetry in the size of HG, with larger volumes in the left than in the right hemisphere^27,78,79^, especially in men^80^. We have replicated this finding using our automated TASH toolbox in a large dataset, further validating our method. TASH can be extended to a wide range of applications. For example, initiatives exist to study the development of the auditory cortex and of other brain structures more generally, from infancy to adulthood^23,69,81,82^, and to study the brains of bilingual adults^13^ and children^83^. Molecular genetic studies have also been done in search of genes which affect auditory cortex structural features^25,84^, and heritability studies have explored the possible contribution of auditory cortex morphology to dyslexia^85^. Our method will allow to perform further such studies in much larger sample sizes using a comparable approach across datasets, allowing for more robust and generalizable findings.

Other applications of our method include further exploration of relationships between auditory cortex structure and basic auditory processing^5,12,86–88^ as well as with higher-level linguistic or musical processing and expertise^8,12,13,39,89,90^. Clinical applications include extension and replication of studies having examined the auditory cortex structure in dyslexia^21,22,91^, in deafness^72,92,93^, in autism^82^ and in schizophrenia^30,94,95^, the latter likely in relation to auditory hallucinations^96^. Our approach can also be adapted for auditory cortex feature selection in the non-human primate brain^97,98^.

TASH allows finer auditory cortex segmentation than is currently possible using existing automated software, with the option of selecting specific auditory cortex subregions and combinations thereof. Furthermore, given the automated nature of our method, it is particularly well suited for examining possible structural plasticity effects longitudinally in the same participants, because TASH’s fine and reproducible feature selection should allow for greater sensitivity to possible experience-dependent structural change over time. Perspectives for new developments of TASH include the implementation of regular updates and improvements to this toolbox in order to adapt and optimize its performance in the context of the changing and variable demands that will likely arise in relation to the anatomical measures required to better explain brain structural phenotypes. For example, next steps include extending TASH for the automated extraction and quantification of the *shape* of the transverse temporal gyrus/gyri (i.e. including the number and type of gyri), in order to allow the automated exploration of gyrification differences in health and disease. Moreover, given evidence for higher myelination within gray matter regions of the PAC^36,37,99^, future developments of TASH can include extensions allowing the integration of data from different imaging modalities (e.g. myelin mapping^36^, functional tonotopic mapping^4,36^ and diffusion-weighted MRI), together with priors derived from cytoarchitectonic atlases^35,78^, with the goal of localizing PAC rather than landmark-based features corresponding to HG.

## Supplementary information

Supplementary Figure 1.

## Data Availability

The computational code developed, and the data generated and/or analyzed during the current study are available from the corresponding author upon reasonable request. The TASH code is available at GitHub (https://github.com/TASH2019/TASH).
