# Supplementary material for: TASH: Toolbox for the Automated Segmentation of Heschl’s gyrus
Source: Sci Rep. 2020 Mar 3;10:3887. doi: 10.1038/s41598-020-60609-y (PMC7054571; doi:10.1038/s41598-020-60609-y)
Supplement: Supplementary file 1 — Supplementary Figure 1. [file 41598_2020_60609_MOESM1_ESM.docx]

**TASH: Toolbox for the Automated Segmentation of Heschl’s gyrus**

***Josué Luiz Dalboni da Rocha^a,*^, Peter Schneider^b,c^, Jan Benner^b,d^, Roberta Santoro^a^, Tanja Atanasova^e^, Dimitri Van De Ville^f,g^, Narly Golestani^a^***

*a. Brain and Language Lab, Department of Psychology, Faculty of Psychology and Educational Sciences, University of Geneva, Switzerland.*

*b. Department of Neurology, Section of Biomagnetism, University Hospital Heidelberg, Germany*

*c. Department of Neuroradiology, University Hospital Heidelberg, Germany*

*d. Division of Diagnostic and Interventional Neuroradiology, Department of Radiology, University of Basel Hospital, Basel, Switzerland*

*e. Faculty of Psychology and Educational Sciences, University of Geneva, Switzerland.*

*f. Medical Image Processing Lab, Institute of Bioengineering, École Polytechnique Fédérale de Lausanne, Switzerland.*

*g. Department of Radiology and Medical Informatics, University of Geneva, Switzerland*

Corresponding author*:

Josue Luiz Dalboni da Rocha, PhD

Josue.Dalboni@unige.ch

Phone: +41 223795866

ORCID: 0000-0001-8939-6395

**Supplementary Information**


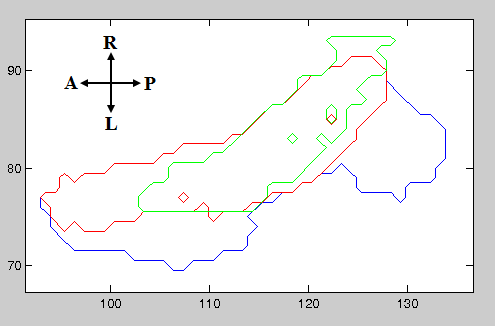


**a.**


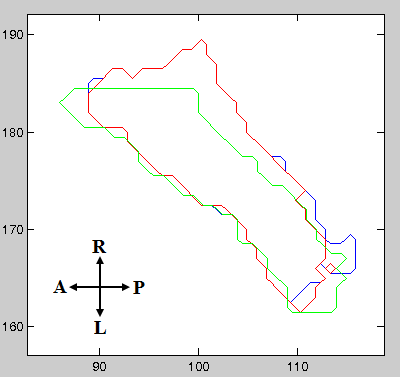


**b.**

**Supplementary Fig. S1** Visual comparison (vertical axis superposed) among manual, TASH and FreeSurfer segmentation results for left (**a**) and right (**b**) HG of a representative participant. Labels are projected in 2 dimensions onto the horizontal plane, showing the overlay of manual delineation (green), TASH label (red) and FreeSurfer label (blue). A: Anterior, P: Posterior, L: Left, R: Right.
